# Early queen joining and long‐term queen associations in polygyne colonies of an invasive wasp revealed by longitudinal genetic analysis

**DOI:** 10.1111/eva.13324

**Published:** 2021-11-30

**Authors:** Giulia Scarparo, Madison Sankovitz, Kevin J. Loope, Erin Wilson‐Rankin, Jessica Purcell

**Affiliations:** ^1^ Department of Entomology University of California Riverside Riverside California USA; ^2^ Department of Fish and Wildlife Conservation Virginia Tech Blacksburg Virginia USA

**Keywords:** polygyny, RADseq, social insects, social transition, *Vespula pensylvanica*, yellowjackets

## Abstract

Invasive social insects rank among the most damaging of terrestrial species. They are responsible for extensive damage and severely threaten the biodiversity of environments where they are introduced. Variation in colony social form commonly occurs in introduced populations of yellowjacket wasps (genus *Vespula*). In particular, invasive colonies may contain multiple queens (i.e., polygyne) and persist several years, while in the native range, the colonies are usually annual and harbor a single queen (i.e., monogyne). In this study, we used genome‐wide loci obtained by double digest restriction site‐associated DNA sequencing (RADseq) to investigate the genetic structure and queen turnover in colonies of the western yellowjacket, *Vespula pensylvanica*, in their introduced range in Hawaii. Of the 27 colonies monitored over four months (October–January), 19 were polygyne and already contained multiple queens on the first day of sampling. Contrary to previous speculation, this finding suggests that polygyny often arises early in the annual colony cycle, before the production of new queens in the fall. Furthermore, polygyne colonies exhibited a prolonged average lifespan relative to those headed by a single queen. As a result, there is no clear window during which colony eradication efforts would be more effective than upon first discovery. The relatedness among nestmate queens was slightly above zero, indicating that these colonies are generally composed of nonrelatives. The queen turnover within each colony was low, and we detected some full‐sibling workers sampled up to four months apart. Finally, we did not detect any population structure among colonies, suggesting that queens disperse up to several kilometers. Taken together, our results provide the first insights into the requeening dynamics in this invasive and incipiently polygyne population and illuminate the early establishment of multiple long‐lasting queens in these damaging colonies.

## 1 INTRODUCTION

Social insects colonize diverse ecosystems and are considered among the most ecologically dominant terrestrial animals. This exceptional success is partly due to the cooperation resulting from eusocial organization (Suarez et al., 2008), which enables groups to respond dynamically to novel or changing environmental conditions, forage efficiently, and defend against predators, along with many other benefits of group living (Moller, 1996). One of the manifestations of social insects’ ecological success is their overrepresentation among widespread invasive insects (Moller, 1996; Rust & Su, 2012; Suarez et al., 2008; Toth et al., 2019). Social insect species often undergo dramatic behavioral and phenotypic modifications following their introduction to novel habitats that may increase their invasion success and impact on the invaded ecosystems (e.g., Urban et al., 2007; Wilson et al., 2009). As a result of this combination of factors, invasive ants, wasps, bees, and termites are responsible for substantial native biodiversity losses and economic damage worldwide (Beggs et al., 2011; Holway et al., 2002; Kenis et al., 2009; Lowe et al., 2000; Rust & Su, 2012; Vargo & Husseneder, 2009).

The invasive yellowjacket species *Vespula germanica*, *V*. *pensylvanica*, and *V*. *vulgaris* have been the focus of numerous studies during the past few decades due to their substantial impact on native invertebrate and vertebrate communities (Beggs et al., 2008; Harrop et al., 2020; Lester & Beggs, 2019; Masciocchi & Corley, 2013; Wilson et al., 2009). These wasps are exceptionally destructive, especially because they invaded remote islands (Courchamp et al., 2003), which lack substantial eusocial fauna capable of ecologically counteracting invaders (Don, 2007; Wilson, 1996). *Vespula germanica* and *V*. *vulgaris* are both native to the Palearctic and were introduced in temperate regions around the world (Australia [Smithers & Holloway, 1977; Spradbery, 1973], New Zealand [Thomas, 1960], South Africa [Whitehead & Prins, 1975], Argentina [D’Adamo et al., 2002; Edwards, 1976; Peña et al., 1975], etc.). They are best known to be among the few insect invaders having a significant impact on New Zealand's native ecosystems (Brockerhoff et al., 2010). In New Zealand, they compete with the native fauna for honeydew resources (Beggs & Wardle, 2006) and reduce native invertebrate prey species populations (Beggs, 2001). Native to western North America, the western yellowjacket *V*. *pensylvanica* was first documented on the island of Kauai in 1919 and subsequently introduced multiple times to six of the eight main Hawaiian Islands (Chau et al., 2015; Gambino et al., 1990). While these invasive populations exhibit genetic differentiation among islands in the Hawaiian archipelago, all have a reduced allelic richness relative to native populations (Chau et al., 2015). In Hawaii, *V*. *pensylvanica* preys upon a wide range of arthropods, of which about 66% are endemic taxa (Beggs et al., 2011; Gambino, 1991, 1992; Wilson et al., 2009). Like some of its congeners, *V*. *pensylvanica* displays an annual life cycle throughout most of its native range, with colonies only containing one functional queen (monogyne) entirely responsible for producing offspring (Akre et al., 1981; Hanna, Cook, et al., 2014). However, recently, invasive populations of western yellowjackets have shifted from an annual monogyne social organization to a polygyne social organization. In up to 20% of cases, these polygyne colonies become perennial and can persist for as many as three years, reaching colony size orders of magnitude larger than colonies from the native range (Gambino, 1991; Goodisman et al., 2001; Hanna, Cook, et al., 2014; Wilson et al., 2009). Within invasive polygyne colonies, the mean relatedness among nestmate workers is low, suggesting that unrelated queens coexist in the same colony (Hanna, Cook, et al., 2014). Similar social transitions to polygyny have been observed in introduced populations of *V*. *germanica* (Goodisman et al., 2001).

This shift in social organization suggests that colonies in the invasive range regularly accept new queens, while colonies in the ancestral population rarely do so (Hanna, Cook, et al., 2014). The causes of the transition in the western yellowjacket *V*. *pensylvanica* remain unknown, although this species appears to be naturally inclined to polygyny due to a weak nestmate discrimination behavior (Loope et al., 2018). Occasional observations of polygyny in the native range (Ratnieks et al., 1996; Visscher & Vetter, 2003) are consistent with this hypothesis. Studying the invasive Hawaiian populations of *V*. *pensylvanica* offers the opportunity to investigate the mechanisms involved in the transition to polygyny and better understand the dynamics of the invasion. Formalizing this knowledge will lay the foundations for effective long‐term management. Despite their ecological and economic impacts, we know very little about the timing of queen production, the frequency and timing of queen acceptance and queen turnover, or the duration of queen reproduction in this species. Most newly established nests become detectable due to an increase in forager activity in August and September (Gambino & Loope, 1992). Production of new queens typically begins in October and November for Hawaiian populations (Gambino, 1991).

Using a population genetic approach with repeated sampling of focal colonies in Hawaii Volcanoes National Park, we investigate changes in the genetic structure of *V*. *pensylvanica* colonies over four months (October–January). Our observation period coincides with the time of the year that we expect colonies to either grow and accept new queens if they are on a perennial trajectory or senesce if they are on an annual trajectory. We carried out our analyses using double digest restriction site‐associated DNA sequencing (ddRADseq), which generated thousands of loci spanning the entire genome. We assessed the queen number and queen turnover within each colony over time and evaluated whether more persistent colonies reliably harbor more queens. Finally, we investigated the spatial genetic structure of the colonies, the relatedness of queens that co‐occur within polygyne colonies, and the relatedness among queens from different colonies. Taken together, our results provide novel insights into the requeening dynamics in this invasive and incipiently polygyne population.

## 2 METHODS

### 2.1 Field sampling

In August–September of 2017, we identified a total of 54 active yellowjacket colonies along Chain of Craters and Hilina Pali Roads in Hawaii Volcanoes National Park (Volcano, HI). Of these, we could safely and consistently sample 27 colonies. Approximately once per month from October 1, 2017, until January 28, 2018, we sampled up to 10 workers entering or exiting the nest entrance of each colony with sufficient activity and placed these in 100% ethanol. We limited the number of collected workers per colony on each date to 10 to avoid inflicting significant damage on the colony, which would bias our assessment of longevity. We did not collect from colonies with fewer than ~10 worker entrances + exits in a two‐minute count; such low activity counts late in the year often indicate the colony is in decline (Loope & Wilson‐Rankin, 2021). All monitored colonies dropped below this threshold after January 28, 2018, but we continued to monitor activity levels weekly until all activity ceased. We transferred all samples to the University of California, Riverside, for subsequent processing. We conducted all fieldwork and collections under permit HAVO‐2016‐SCI‐0050.

Colony survival was monitored as part of another study on the ecological correlates of longevity (for details, see Loope & Wilson‐Rankin, 2021). Briefly, colony traffic was monitored every 7–21 days until the colony was scored as “zero traffic” two times in a row. Average colony size was predicted according to the equation of Malham et al. (1991). Colony longevity was estimated as the number of days survived past September 1, 2017. We used the Mayfield 40% method (Johnson, 1979) to account for the interval between the last time each nest was observed to be active and the first time it was observed dead. A subset of these colonies was provided diet subsidies in the form of frozen honeybees or passively heated with perspex cones surrounding the nest entrance (Loope & Wilson‐Rankin, 2021). Neither treatment was found to affect colony longevity nor influence queen number in this study (analyses not shown).

### 2.2 Library preparation

In total, we collected 760 individual wasp workers across 27 colonies and five sampling dates. We extracted DNA from the head of each worker using a modified version of the Qiagen DNEasy protocol for insect tissue (Qiagen Inc). Deviations from the protocol include the substitution of 70% ethanol for AW2 buffer and the use of alternatively sourced spin columns (bpi‐tech.com). We constructed double digest restriction site‐associated DNA sequencing (RADseq) libraries using a customized protocol (Brelsford et al., 2016). In brief, we digested DNA with the enzymes SbfI and MseI, ligated adapters (including 96 unique in‐line barcoded adapters to the SbfI end), removed small DNA fragments using Sera‐Mag Speed Beads (Thermo Fisher Scientific), ran quadruplicate PCRs for each individual (adding a unique Illumina index sequence for each plate), pooled the four replicates and added additional primer mix and dNTP for one final PCR cycle to minimize single‐stranded DNA fragments, ran each sample on a gel to ensure that the library was successful, and pooled individual libraries. We included one negative control per plate to ensure that there was minimal contamination. We ran a final bead clean‐up to remove small DNA fragments from the pooled library before sequencing. The library was sequenced on an Illumina HiSeq X10 lane by Novogene Inc. This protocol generates thousands of markers spanning the genome.

SNP datasets such as this are becoming popular for parentage and social structure analyses, given the relative ease of obtaining genotypes for hundreds of variable loci, and their power is typically greater than a traditional microsatellite approach (Attard et al., 2018; Flanagan & Jones, 2019; Lemopoulos et al., 2019). Furthermore, having a large number of genetic variants should be particularly useful in populations that may have low genetic variation due to a recent genetic bottleneck, as expected in invasive populations (Lemopoulos et al., 2019). Finally, accumulating evidence suggests that social insects frequently have unequal patterns of genetic variation across the genome, which could result, for example, from the presence of chromosome‐spanning supergenes (e.g., in ants: Lagunas‐Robles et al., 2021; Purcell et al., 2014; Wang et al., 2013) or unorthodox reproductive modes (e.g., Eyer et al., 2019; see also Beekman & Oldroyd, 2008; Kuhn et al., 2020). While such patterns have not been described in social wasps to our knowledge, using genome‐wide, reduced representation sequencing methods increases our ability to detect and account for any unexpected variation across the genome.

### 2.3 Bioinformatics

We demultiplexed individuals using the process_radtags function in Stacks (Catchen et al., 2013) and merged overlapping paired‐end reads with PEAR (Zhang et al., 2014). Then, we aligned reads to the *V*. *pensylvanica* genome (Harrop et al., 2020) using BWA‐mem (Li & Durbin, 2009) and called variants using BCFtools mpileup (Li et al., 2009). A total of 43 individuals distributed among colonies were excluded from the analysis at this stage, either because the library preparation failed and we did not pool the individual or because they exhibited high levels of missing data, indicating sequencing failure. We filtered variants in VCFtools as follows: we initially removed uncertain genotype calls using a combination of the minGQ filter (minimum genotype quality score of 20) and the minDP filter (minimum sequencing depth of 1) in parallel. We then removed loci with more than 20% missing data (max‐missing 0.8) and with a minor allele frequency of <5% (maf 0.05). To obtain independent loci necessary for inferring colony family structure, we used PLINK 1.9 (Purcell et al., 2007). At first, we calculated the squared allele count correlation (*r* ^2^) for each pair of SNPs and plotted the values on a histogram to find the *r* ^2^ cut‐off value that excludes the tails of the distribution. We then removed one locus from each pair of loci with *r* ^2^ > 0.1 within a 200 kb sliding window (Andrews et al., 2018). This filtering resulted in a dataset containing 346 loci in 717 individuals from 27 different colonies (mean read depth per individual = 123.99).

We also produced a second dataset merging one individual from each of the 27 colonies and one individual from the 9 *V*. *pensylvanica* colonies analyzed in M. Sankovitz et al. (unpublished data). The latter nine colonies are distributed in Volcano Village, with the closest colony (CRTWP24) located at a distance of 4–10 km from our focal colonies and the farthest colony (TAWP4) 15–23 km away from the focal colonies. From each colony, we retained the individual with the least missing data. For this second dataset, we filtered variants in VCFtools using the same minGQ, minDP, and max‐missing filter values as above. We included only sites with minor allele count greater than or equal to 2 (mac 2). We then used PLINK 1.9 (Purcell et al., 2007) to filter one locus of each pair of SNPs in a sliding 200 kb window using a threshold *r* ^2^ > 0.25 (Andrews et al., 2018). This filtering resulted in a dataset containing 458 loci in 36 individuals from 36 different colonies.

### 2.4 Analysis

We used COANCESTRY 1.0.1.10 (Wang, 2011) to determine relatedness among workers (using Wang [2002] estimator). A recent literature review and simulation study confirmed that relatedness estimates tend to be downward biased, yet more precise, in SNP‐based datasets with hundreds or thousands of loci compared to microsatellite‐based datasets with fewer loci (Attard et al., 2018). This downward bias in relatedness is prevalent in RADseq datasets due to a combination of genotyping error, allelic dropout, and missing data. Given the known biases in datasets like ours, we visualized the overall relatedness values of nestmates versus non‐nestmates in our samples, shown with a scaled density plot produced in R (R Core Team, 2020) using the function “ggplot” (package ggplot2, Wickham, 2009). This approach clearly identifies the relatedness levels of full‐ and half‐sibs in our dataset, despite deviations from pedigree‐based predictions. In addition, we examined the average pairwise relatedness values of nestmate workers across sampling time points.

To assign putative parentage to groups of nestmate workers over time, we used the program COLONY v. 2.0.6.5 (Jones & Wang, 2010), using the full‐likelihood model. COLONY produced estimates of the number of queens and fathers contributing to reproduction in each colony. For the only polygyne colonies, we produced a stacked bar plot showing the queens’ contribution to worker production within each colony. COLONY also provided probability estimates for inferred parental genotypes at each locus when the genotype is uncertain. To estimate relatedness among queens, we averaged relatedness estimates across 100 samplings from estimated queen genotypes using “related” package in R based on COANCESTRY (Pew et al., 2015), with each queen's potential genotype at each locus sampled in proportion to their relative probability estimated by COLONY. We then compared the average pairwise relatedness values of nestmate queens and non‐nestmate queens, displayed with a scaled density plot.

To assess whether the estimated number of queens per colony correlated with estimated colony longevity, we calculated the Spearman correlation using the “ggscatter” function (package ggpubr, Kassambara, 2020). We calculated this relationship based on the total number of queens detected over our full sampling period, both for all the colonies and for polygyne colonies only. Finally, to avoid bias based on the dependency of queens’ number to the analyzed number of workers, we ran another Spearman correlation based on the number of queens detected on the first day that we sampled each colony, which is based on the analysis of 8–10 workers per colony.

We assessed whether the genetic structure of our focal population that included 27 colonies within Hawaii Volcanoes National Park varies geographically and over the four‐month time window or with colony social form using STRUCTURE 2.3.3 (Pritchard et al., 2000). Applying the admixture model, we ran the model 10 times for each *K*‐value from 1 to 10, with a burn‐in period of 50,000 iterations, followed by 100,000 iterations per test. We inferred the most probable *K* value using the Δ*K* “Evanno method” (Evanno et al., 2005) using STRUCTURE HARVESTER Web v 0.6.93 (Earl, 2012). For a given set of simulations for each *K*, we used CLUMPP v 1.1.2b to align the 10 replicate runs (Jakobsson & Rosenberg, 2007). We then used Distruct v 1.1 to visualize the results of the clustering process (Rosenberg, 2004).

Finally, to assess if *V*. *pensylvanica* exhibits more population structure at a larger spatial scale (up to ~23 km), we ran a second STRUCTURE analysis with the same parameters stated above, comparing one worker per colony from each of our samples with one worker pupa per colony analyzed in M. Sankovitz et al. (unpublished data). We used STRUCTURE HARVESTER Web v 0.6.93 to infer the most probable *K* value, and we aligned the 10 replicate runs using CLUMPP v 1.1.2b. We then visualized the bar plot using Distruct v 1.1 (Rosenberg, 2004).

## 3 RESULTS

**FIGURE 1:**
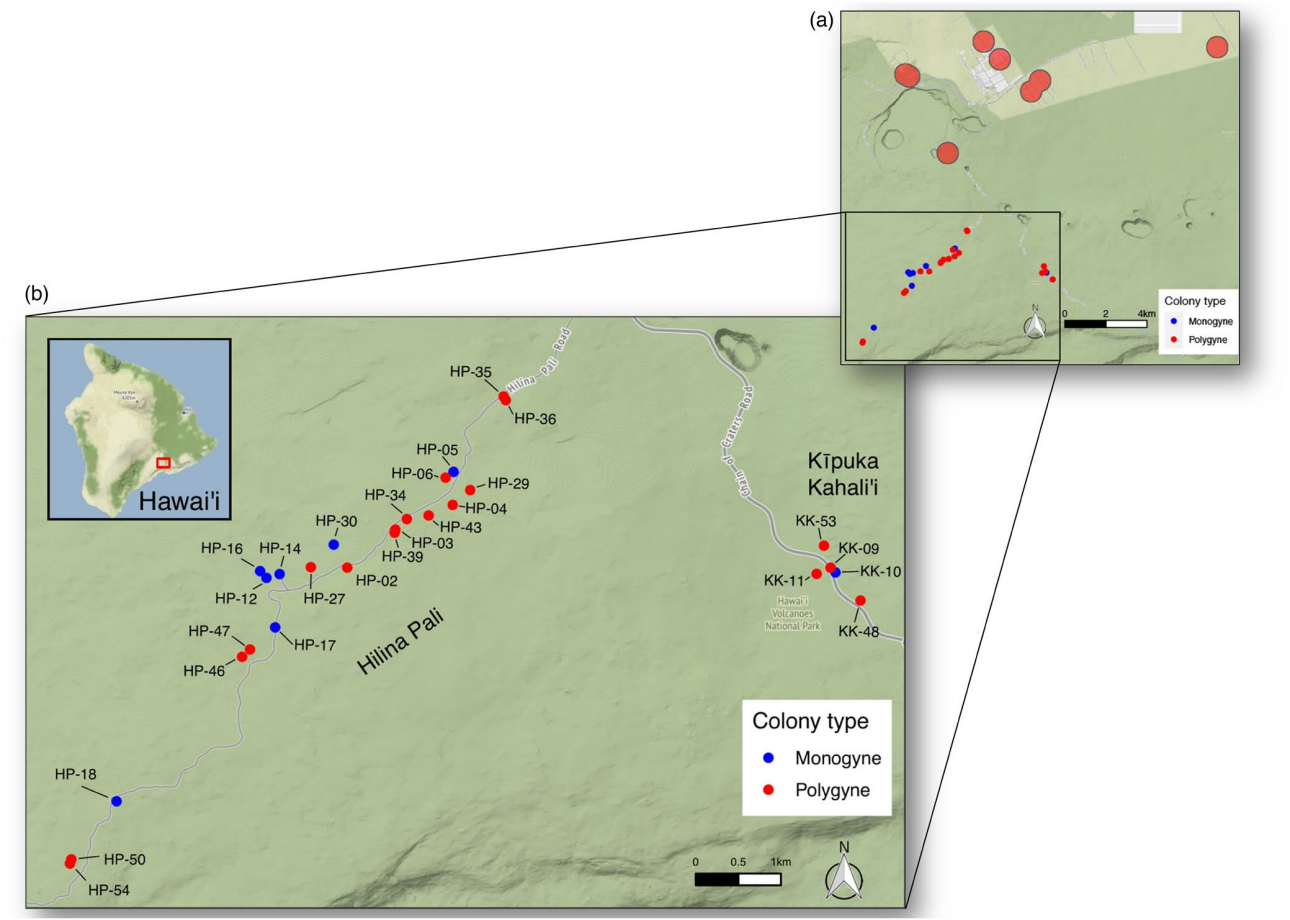
Distribution map showing the 27 focal colonies analyzed in this work (red and blue dots) and the 9 colonies analyzed in Sankowitz et al. (pink shaded circles show an approximate position to preserve the privacy of property owners) (a). The 27 focal colonies were distributed along Hilina Pali Rd and Chain of Craters Rd in Hawaii Volcanoes National Park (b). We identified both monogyne (8 of 27, blue dots) and polygyne (19 of 27, red dots) colonies in our study area

**FIGURE 2:**
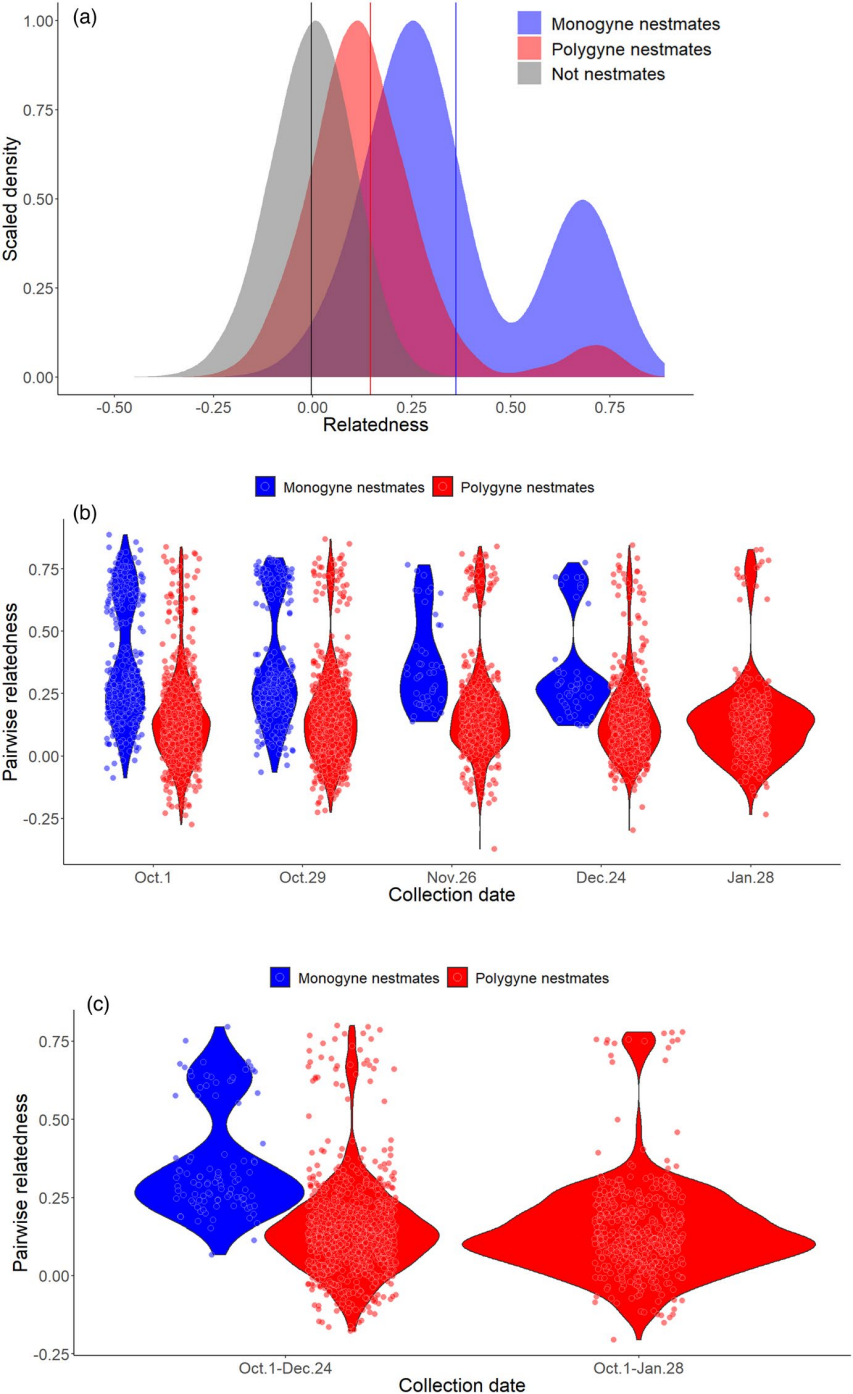
Density plots (a) reveal that monogyne colonies (in blue) exhibit an average within colony worker relatedness of 0.36 (blue vertical line), while the average relatedness of polygyne (in red) colonies is 0.15 (red vertical line). Overall monogyne colonies harbor a mix of full siblings and half siblings (bimodal distribution of relatedness values, peaks at ~0.70 for full sibs and ~0.20 for half‐sibs reflect the downward bias typical of relatedness estimates based on RADseq markers; Attard et al., 2018). Points of the two violin plots (b,c) show within‐colony pairwise relatedness values between nestmate workers sampled on the same date (b) and between dates (c). The difference in the relatedness of workers in monogyne and polygyne colonies across our longitudinal sampling remained similar through time (b), although most of the monogyne colonies declined after Oct 29, and none persisted through Jan 28. We detected full siblings in samples collected 3–4 months apart in both monogyne and polygyne colonies (c)

In this study, we investigated colony dynamics in an invasive Hawaiian population of *V. pensylvanica*. Of the 27 colonies genotyped, 29.6% (8 out of 27) were monogyne with an average colony size of 850 (± 235) estimated from traffic rates in early September (Figure 1; Table 1). The remaining polygyne colonies had an average size of 1843 (±636) and harbored offspring produced by 2–8 queens. We found an average relatedness among workers from monogyne colonies of 0.36 (±0.22) and an average relatedness among workers of 0.15 (±0.18) in polygyne colonies (Table 1, Figure 2a, Figure S1a,b). As expected, the observed modal peak values corresponding to full and half siblings are slightly lower than we would expect based on pedigree‐based inference, in which full siblings should have an average relatedness of 0.75 and half siblings an average relatedness of 0.25 (Figure 2a). However, previous studies demonstrate that estimating relatedness from RADseq loci tends to be precise in relative terms but causes calculated relatedness values to be downwardly biased compared to theoretical predictions (Attard et al., 2018).

**TABLE 1:** Overview of colony social structure and longevity

Colony	Sampling (days, N° of workers used for the analyses after filtering)	Longevity (days from Sept 1)	Estimated colony size (# of workers)	COLONY result					Worker to worker mean relatedness	Queen to queen mean relatedness
				Estimated number of queens	Estimated number of newly adopted queens (included queens attributed to <15% of worker production)	Estimated number of queen losses (included queens attributed to <15% of worker production)	Estimated number of fathers per colony	Observed paternity^a^		
HP‐02	2, 16	136	2644	5	‐ (1)	‐ (1)	14	3.7	0.12	0.08
HP‐03	4, 36	172	2160	8	‐ (2)	1 (4)	24	7.5	0.16	0.08
HP‐04	3, 29	164	2257	5	‐ (1)	—	21	5	0.12	0.11
HP‐05	1, 10	101	903	1	—	—	2	2	0.54	—
HP‐06	5, 48	185	1451	7	‐ (2)	‐ (4)	34	8	0.11	0.14
HP‐12	2, 19	157	870	1	—	—	5	5	0.27	—
HP‐14	2, 20	136	1064	1	—	—	5	5	0.31	—
HP‐16	1, 8	58	774	1	—	—	5	5	0.27	—
HP‐17	4, 40	150	322	1	—	—	4	4	0.37	—
HP‐18	1, 10	94	1064	1	—	—	4	4	0.43	—
HP‐27	5, 48	213	645	6	1 (2)	2 (5)	24	5.5	0.13	0.17
HP‐29	1, 9	101	1419	2	—	—	4	3	0.18	0.01
HP‐30	2, 20	86	935	1	—	—	3	3	0.38	—
HP‐34	2, 20	157	2321	6	1 (2)	‐ (1)	11	2.25	0.13	0.07
HP‐35	5, 47	220	2128	8	‐ (4)	1 (2)	37	8.3	0.08	0.14
HP‐36	1, 9	52	1322	4	—	—	9	2.7	0.01	0.02
HP‐39	1, 10	108	1161	5	—	—	8	2	0.03	0.03
HP‐43	4, 39	164	2547	3	—	‐ (1)	8	3	0.27	0.16
HP‐46	2, 18	94	967	2	—	—	5	2.5	0.20	0.13
HP‐47	5, 47	192	2031	4	—	1 (1)	16	4	0.22	0.21
HP‐50	3,29	192	2708	8	1 (5)	1 (3)	26	8.5	0.07	0.05
HP‐54	4, 40	164	1322	8	3 (5)	1 (4)	32	5.5	0.09	0.09
KK‐09	3, 28	164	2225	5	—	—	15	3.7	0.18	0.09
KK‐10	2, 19	108	870	1	—	—	3	3	0.38	—
KK‐11	4, 39	178	1128	6	1 (1)	1 (2)	12	2.5	0.17	0.12
KK‐48	4, 39	172	1999	6	‐ (1)	‐ (2)	25	7.5	0.17	0.17
KK‐53	2, 20	108	2579	6	1 (1)	‐ (1)	14	2.4	0.05	0.03

Notes

This table shows the number of sampling days per colony, the number of workers used for genetic analysis, the estimated colony size, the colony longevity, the estimated number of queens and fathers per colony, the number of estimated requeening events and queen losses in each colony among queens mothering at least the 15% of worker production in our sample, the observed paternity, and mean relatedness among nestmate workers and nestmate queens. The estimated number of queens and fathers and estimated queen adoptions and queen losses were inferred using COLONY. The number of queen adoptions was calculated by counting the number of queens detected for the first time after the first sampling day within each colony. The number of queen losses was estimated by counting the number of queens detected on early sampling dates, but absent from later sampling dates. Some queens were inferred through a few workers in our sample (<15% of workers within the sample), and so their apparent gain or loss has less support. These queens are shown in parentheses in both the gains and losses columns. For more details, see Table S1.

^a^ The observed paternity was calculated only for those queens parenting two or more workers.

In line with a concurrent study (M. Sankovitz et al., unpublished data), nestmate queens varied substantially in their contributions to worker production in these colonies (see also Figure S2). For some of our analyses (observed paternity and queen turnover), we focus on queens that produced at least 15% of the workers that we sequenced. Hereafter, these queens are distinguished as “queens contributing substantially to worker production.” We have limited power to assess the mate number and longevity of queens represented by only 1–2 workers in our dataset.

All queens parenting more than a single worker in our sample appeared to be polyandrous. The average observed paternity for polygyne colonies was 4.6 (±2.31) and 3.9 (±1.12) for monogyne colonies (Table 1). Despite the 15% threshold, paternity estimates for polygyne colonies may still be biased. For those colonies sampled only 1–2 times before decline, we have sometimes included queens that only mothered 2–5 workers, for which we, therefore, may not have a complete picture of observed paternity.

**FIGURE 3:**
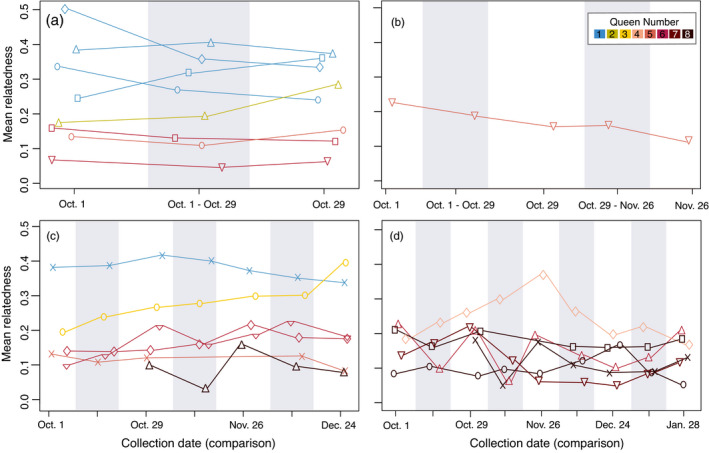
Mean relatedness among nestmate workers collected on the same date (white panels) or on consecutive collecting dates (grey panels) is consistent through time in most colonies. Colonies that persisted to be sampled at least twice are shown and are color coded by the inferred number of queens present. Colonies that were sampled at the beginning and end of October only (a), in October–November (b), and in October–December (c) exhibited little fluctuation in relatedness both within and between sampling dates, indicating that queen turnover is rare. We noted more fluctuations in several colonies that were sampled from October–January (d). In cases of high queen turnover, we would expect to observe lower relatedness between nestmate workers collected on different dates than within the same day. This pattern was observed, for example, in the colony represented by upward triangles in panel d. In this case, the fluctuation of the average relatedness among days is attributed to variation in queen representation among sampled workers (see full explanation in the results section)

**FIGURE 4:**
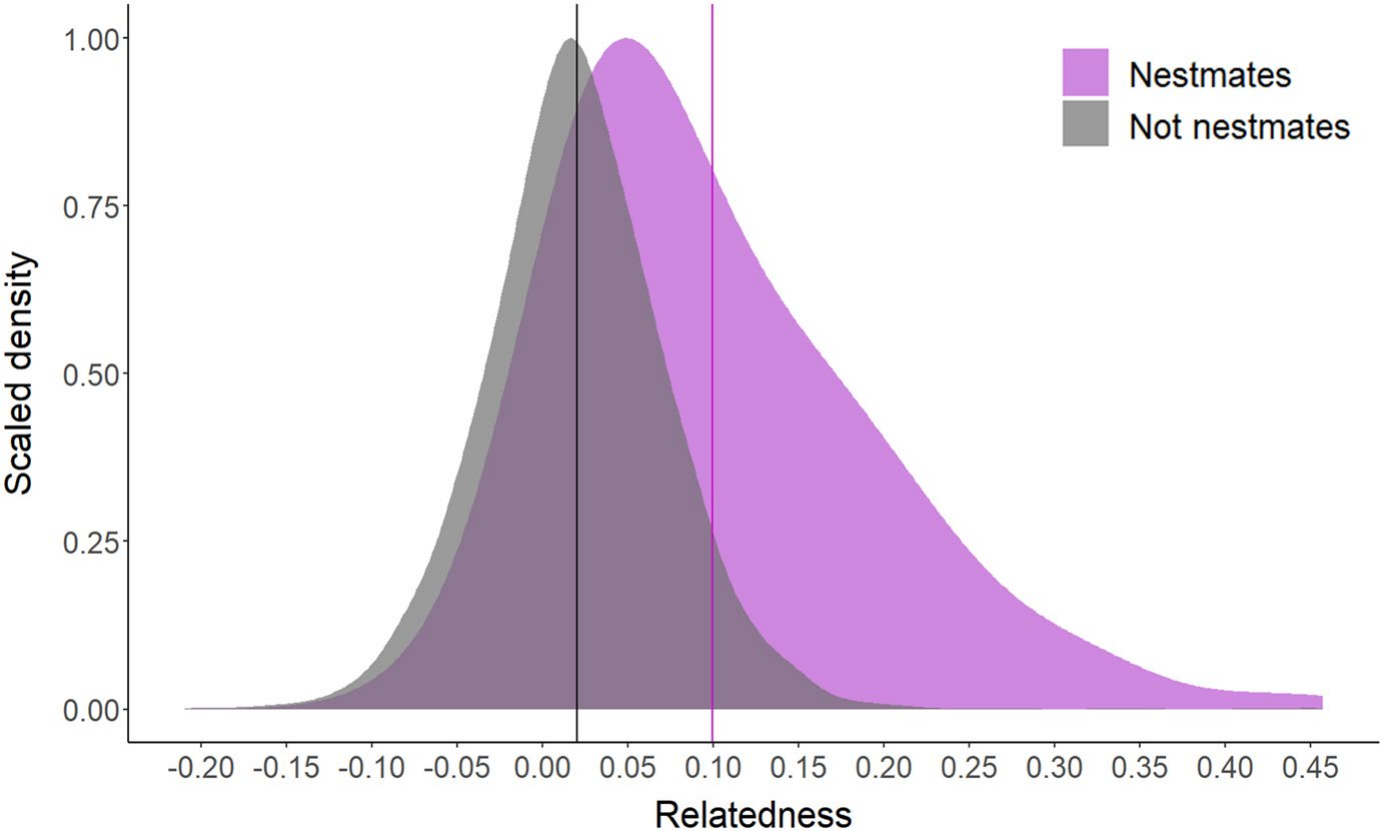
Density plot using kernel density estimates shows that, on average, nestmate queens are only slightly more related to one another (average relatedness = 0.1, purple vertical line) than non‐nestmates (average relatedness value = 0.02, black vertical line). We observe some instances of apparent half sibling queens (right tail) or cousins in shared nests, but most nestmate queens appear to be unrelated

We assessed queen turnover using two complementary methods: We directly assessed the worker parentage through time using the inferences from COLONY (Table 1), and we assessed changes in relatedness within sampling date and between dates for colonies that persisted for at least one month. Apparent queen gains or losses based on COLONY‐inferred parentage were infrequent for queens contributing substantially to worker production (Table 1). We saw a similar pattern of stability in nestmate relatedness among workers sampled on the same date or on consecutive sampling dates (Figures 2b and 3). Moreover, we detected some full sibling workers sampled on days up to four months apart (Figure 2c). This indicates that some queens persist and reproduce in polygyne colonies for many months [in comparison to adult *Vespula* workers, which typically live for fewer than 20 days (Toth et al., 2016)] and that polygyny is stable within a colony. There are several exceptions to this general pattern. In some such cases, offspring of certain queens were not sampled at intermediate time points but were sampled early and late in our observation period. For instance, the colony HP‐27 showed an apparent nestmate relatedness fluctuation ranging between 0.15 and 0.05 (Figure 3d, red upward triangles). This trend is ascribable to an apparent decrease from four to two queens mothering offspring from October 1 to October 29, in this case, consistent with three queen losses and one queen adoption (Table S1). However, offspring of the same queens observed on October 1 were found again on November 29, clearly showing that at least some observed losses actually stem from sampling bias. As a result, we cannot distinguish between real queen gains and losses as opposed to those that reflect sampling biases, particularly for queens that are not well represented in our sample of workers. However, we can still draw inferences from the turnover among queens that substantially contribute to worker production, as these are less likely to be markedly influenced by sampling bias. Despite the persistence and apparent prolonged mutual tolerance of multiple queens in these nests, nestmate queens were not highly related, with an average relatedness of 0.1 (±0.09; Figure 4).

**FIGURE 5:**
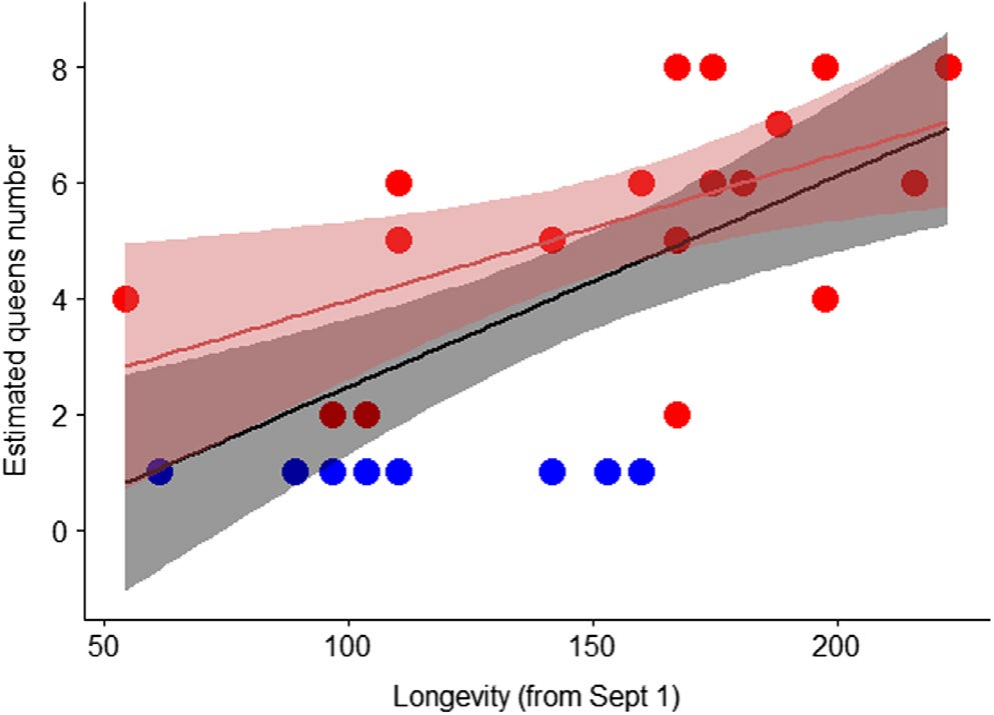
Colonies with greater numbers of queens persist longer than those with one or few queens. The black line shows the positive correlation between queen number and colony longevity in all colonies (monogyne and polygyne; Spearman correlation, *r* = 0.69, *p* < 0.0001). The red line shows the correlation in only polygyne colonies (*r* = 0.6, *p* = 0.0068). Blue dots represent monogyne colonies, red dots show polygyne colonies

We observed a strong correlation between the number of queens present in a colony and that colony's persistence into January and February (all colonies: Spearman correlation, *r* = 0.69, *p* < 0.0001; only polygyne colonies: *r* = 0.6, *p* = 0.0068; Figure 5). This correlation is not due to dynamic acceptance of new queens. Instead, colonies that already have many queens on October 1 are likely to persist longer than those colonies with few queens (Spearman correlation, *r* = 0.48, *p* = 0.011, Figure S3).

STRUCTURE analyses revealed minimal population genetic structure at the scale of our study area, wherein colonies were positioned up to 5 km apart (Figure S4a,b). Moreover, we found no difference in the population structure of monogyne and polygyne colonies within our study area. We also did not find within‐colony genetic differentiation over four months. When we assessed the genetic structure of our focal colonies plus nine colonies located outside of our study area, which were collected in the same year for an independent study (M. Sankovitz et al., unpublished data), we observed moderate population structure (Figure S4c).

## 4 DISCUSSION

Although the ecological invasion impacts of *V. pensylvanica* in the Hawaiian Islands have been previously investigated, to date, the drivers and dynamics underlying the social transition from monogyny to polygyny remain obscure. Our study illustrates the genetic structure and colony dynamics of invasive *V*. *pensylvanica* in Hawaii Volcanoes National Park during a four‐month time span. Seventy percent of the colonies we detected were polygyne. The relatedness among nestmate workers and nestmate queens was only slightly elevated above the population average, indicating that these colonies are predominantly composed of nonrelatives. We saw infrequent evidence of queen turnover (among queens contributing substantially to worker production) or requeening events, suggesting that queen assemblages form early in the season and remain remarkably stable as the colonies enter the wet season. The number of queens present in early October (soon after these colonies have sufficient traffic to be readily detectable in the field) is a good predictor of their likelihood to persist into the rainy season (November–March).

Three possible mechanisms may explain the presence of polygyny in vespine colonies: either queens cooperate during colony establishment (i.e., primary polygyny), daughter queens are recruited from within their natal nests, or foreign queens enter established nests (Archer, 2010; Keller, 1995). So far, researchers believed that colony foundation by pleometrosis was unlikely in *Vespula* wasps (Greene, 1991; Hanna, Cook, et al., 2014). However, previous studies generally assumed that joining queens are new reproductives produced in the fall (e.g., Greene, 1991). We observed many polygyne colonies on October 1, prior to when most new queens are produced in the late fall (Gambino, 1991). We found that almost all the queens detected in each colony were unrelated, which aligns with Hanna, Cook, et al. (2014) observation of multiple mitochondrial haplotypes within some of their colonies sampled on a single date. Moreover, none of the detected monogyne colonies adopted queens during our monitoring period. Taken together, these results suggest that the polygyny is not the result of secondary recruitment of daughter queens, as observed for the congener, *V*. *germanica*, experiencing the same social transition (Goodisman et al., 2001). It is possible that colonies are initiated pleometrotically by multiple, unrelated queens, though this, to our knowledge, has never been described in the genus *Vespula*; instead, queens are mutually intolerant during the founding stage, likely due to frequent inter‐ and intraspecific usurpation (Akre et al., 1976, 1977; MacDonald & Matthews, 1981). Another non‐mutually exclusive possibility is secondary polygyny occurring very early in the colony life cycle. Importantly, this implicates late‐flying queens of the same generation as the foundress, which are common into June and July (Gambino, 1991), as the likely source of joiners. This mechanism is also consistent with the apparent weak nestmate discrimination behavior of *V*. *pensylvanica* workers in Hawaii (Loope et al., 2018), which would permit queens to join preexisting colonies without being attacked.

Both polyandry and polygyny are relatively common in social insect colonies, despite the fact that both mating systems reduce nestmate relatedness. Polyandry increases risks of predation and parasitism to queens and increases energy expenditure during the nuptial flight (Crozier & Fjerdingstad, 2001). However, numerous studies have suggested possible advantages of polyandry (Baer & Schmid‐Hempel, 1999; Crozier & Fjerdingstad, 2001; Keller & Reeve, 1994; Mattila & Seeley, 2007), among these: a reduced risk of sperm depletion; an improved division of labor among workers; an increased response of the colony to tolerate different environmental conditions; and an enhanced defense against pathogens. Polygyny leads to similar colony outcomes. In monogyne and monandrous colonies, relatedness among workers is usually very high, with a predicted value of 0.75. However, combined or alone, polygyny and polyandry can dramatically reduce the intracolony relatedness, thus increasing the possibility of intracolony conflicts among nestmates (Crozier & Fjerdingstad, 2001). In polyandrous colonies, half siblings will have a predicted mean relatedness of 0.25, with the average within colony relatedness falling between 0.75 and 0.25, with the relative level depending upon the number of mates. Among Hymenoptera, polygyny and polyandry are usually negatively correlated with each other (Hughes et al., 2008), although the invasive populations of *V*. *pensylvanica* show both reproductive strategies. We found that in monogyne colonies, the average relatedness is 0.36, as a result of multiple mated queens, while in polygyne colonies, the average relatedness is 0.15, as a result of combined polyandry and the coexistence of many unrelated queens. Our pairwise relatedness estimates are slightly lower than those reported by Hanna, Cook, et al. (2014), with peaks centered at ~0.70 for full sibs and ~0.20 for half siblings. Estimates of relatedness tend to be biased downward by due to occasional genotyping errors and allelic dropout in RADseq data, but relatedness values tend to be more precise in relative terms compared to those calculated from microsatellites (Attard et al., 2018).

All *V*. *pensylvanica* queens parenting at least two workers were multiply mated. Polygyne colonies showed an average observed paternity (4.6), which is similar to the observed paternity identified in monogyne colonies (3.9). Referring to the only monogyne colonies, our finding does not differ from the minimum mate number results found by Hanna, Cook, et al. (2014) for the native monogyne colonies in Washington (4.33 ± 1.68), while it is slightly lower than the average mating rate for the invasive monogyne colonies analyzed in the same work (5.4 ± 2.6) (Hanna, Cook, et al., 2014). However, the higher average found by Hanna et al. is due in part to a single colony whose queen mated with 10 males. Excluding this colony from the analysis, the average minimum mate number decreases to 4.25 (±0.5), aligning with our results. Within the *Vespula* genus, polyandry is common (Loope et al., 2014). Eastern yellowjacket *V*. *maculifrons* queens produce a higher number of gynes when mated with many males compared to queens mated with a few males (Goodisman et al., 2007). Recently, it has also been demonstrated that polyandry in the monogynous *V*. *shidai* helps the colony to better resist different pathogens strains rather than colonies founded by single mated queens (Saga et al., 2020).

COLONY analysis shows that queen turnover within each colony was low, although this result reflects mainly the queens contributing substantially to worker production. As *V*. *pensylvanica* queens contribute unevenly to offspring production (M. Sankovitz et al., unpublished data; Figure S2), analysis of 10 workers per colony each month is likely to be insufficient to detect all matrilines, especially those producing the fewest worker offspring. Therefore, we may have missed some requeening events, especially those that occurred in highly polygynous colonies. In parallel, queens already present in the colony since the first day of sampling may have only been detected in our analysis at a later sampling date. In addition, estimates of queen turnover over time may also reflect temporal changes in queen fertility rather than queen mortality, especially in those colonies experiencing strong reproductive skew. If this were the case, our monthly samples would tend to preferentially detect queens close to their reproductive peak, while more queens could be present inside the colonies but with their reproductive peak shifted to another period of the year (Bargum et al., 2007). However, none of the initial eight monogyne colonies ever adopted new queens, and all 19 polygyne colonies retained multiple queens from the colony's first to final sampling day. Taken together, our results suggest that the *V*. *pensylvanica* social form is already fixed by early October and that queen number remains relatively stable.

Our results contrast with findings from other social insects, which show variable queen turnover rates. The serial polygynous wasp, *Ropalidia marginata*, showed frequent queen replacements, up to 10, throughout the year without a clear relation with the colony cycle (Gadagkar et al., 1993). Among *Polistes* wasps, dominance behavior is commonplace, where a single queen dominates the reproduction of the colony. However, episodes of queen replacements by dominant workers were frequently observed after the death or removal of the resident queen (Jandt et al., 2014). Long‐term monitoring of many ant species estimated average annual queen turnover up to 60% (Bargum et al., 2007; Evans, 1996; Pedersen & Boomsma, 1999). This high replacement rate was justified by very short queen lifespan and high mortality or by short‐period reproduction. On the other extreme, queen turnover over several years in *Formica selysi* colonies was absent or very rare (Chapuisat et al., 2004), although it was more readily detectable when the study period was expanded to include more than 10 years of sampling data (Purcell & Chapuisat, 2013).

Until now, it has been assumed that polygyny in *Vespula* wasps is a prerequisite for perennial colonies (e.g., Gambino, 1991), although never clearly demonstrated. In this study, we showed that polygyne colonies hosting many reproductive queens tend to persist longer than colonies with few or just one queen. In contrast, no monogyne colony survived the entire sampling window, and most of them were already in decline by the end of October. Interestingly, we did not find a significant decrease in queen number before the colony extinction, suggesting that polygyne colony decline does not start after the onset of mortality of the reproductive queens but may instead be driven by their reduced oviposition activity or independent environmental factors governing colony success. None of the colonies observed in this study survived until the next summer. Although volcanic activity in early 2018 may have contributed to their final decline during this time, results from similarly monitored colonies in other years suggest that the probability that a given autumn colony survives until the next season is often quite low (~1%) at these sites (Loope & Wilson‐Rankin, 2021). While we can only speculate about the link between perennialism and polygyny elsewhere, it seems possible that the increase in colony longevity that is associated with increasing queen numbers observed here could similarly result in an increase in the probability of perennialism for highly polygyne colonies in environments more suitable to colony overwintering.

The proportion of monogyne colonies that we detected in 2017 (29.6%) was slightly higher than the one found by Hanna, Cook, et al. (2014), 15%), which was based on samples collected from Hawaii Volcanoes National Park in 2008 and 2010. The Hawaiian Islands experience frequent volcanic activity, with significant pyroclastic events of Kilauea having occurred in 2008 (Tilling et al., 2010) and 2018 (Global Volcanism Program, 2018). The observed colonies in this study ceased activity shortly before the lower Puna eruption of Kilauea in 2018. During a subsequent sampling trip in 2019, we observed a density of yellowjacket nests that was substantially higher than the density observed in past years (Rankin, Purcell, and Wilson‐Rankin, personal observation). These yellowjackets are likely to be able to rapidly recolonize suitable habitat following die‐offs due to volcanic gases, although our results suggest that dispersal is most common at a local scale, consistent with the findings of Chau et al. (2015). We do not know if a higher proportion of the newly established colonies found in 2019 were monogyne, although unstable conditions are thought to favor monogyny (Seppä et al., 1995). In general, we speculate that changes in volcanic activity could influence the frequency of monogyne and polygyne colonies in this system.

The perennial phenotype greatly amplifies the impact of invasive yellowjackets because the colonies can reach monstrous sizes, supported by numerous ovipositing queens, which allows them to compete with and prey upon a large diversity of arthropods throughout the year, including endemics (Wilson & Holway, 2010; Wilson et al., 2009). Invasive wasps disrupt plant–pollinator interactions of other introduced (e.g., *Apis mellifera*) and native *Hylaeus* bees (Hanna et al., 2013, 2014). Western yellowjacket nests are typically subterranean, with the entrance concealed within vegetation or at the base of trees or rocks. This makes early detection when colonies are small and more vulnerable almost impossible, leading to pest management challenges. Unfortunately, there does not seem to be a more suitable time of year to eradicate the colonies, other than as soon as colonies are detected, since queen acceptance appears to occur very early in the colony life cycle. Strategies targeting free‐flying queens in early summer, when they are most abundant (Gambino, 1991), could possibly reduce the pool of potential joining queens and influence the frequency of polygyny. The use of fipronil in baited feeding stations has been found to significantly reduce the *Vespula* population in the short and long term (Hanna et al., 2012), though the nonspecificity of both the pesticide and carrion attractant used poses a potential danger for native fauna. There is a great need to improve the species specificity in poison bait attractants to reduce impacts on nontarget species. Moreover, fipronil is not registered for yellowjacket removal, and its use is illegal. The use of long‐range sex pheromones to confuse the males’ ability to find receptive females could be a valid alternative, although pheromonal communication in *Vespula* is likely complex and context‐dependent (Derstine et al., 2017, 2018). However, this area is rapidly developing, and more research is ongoing. Future studies aimed at following perennial colonies for multiple years, determining how long queens live and contribute to reproduction, and comparing phenology between the native and invasive range could improve our knowledge of this species’ social transition and provide better guidance for more efficient management of this pest.

## CONFLICT OF INTEREST

The authors declare no competing interest.

## Supporting information

Fig S1‐S4

Table S1

## Data Availability

Genetic sequences reported herein have been deposited at the NCBI Sequence Read Archive with the accession number PRJNA737151.
