# Supplementary material for: Early queen joining and long‐term queen associations in polygyne colonies of an invasive wasp revealed by longitudinal genetic analysis
Source: Evol Appl. 2021 Nov 30;14(12):2901–14. doi: 10.1111/eva.13324 (PMC8674895; doi:10.1111/eva.13324)
Supplement: Supplementary file 1 — Fig S1‐S4 [file EVA-14-2901-s001.docx]

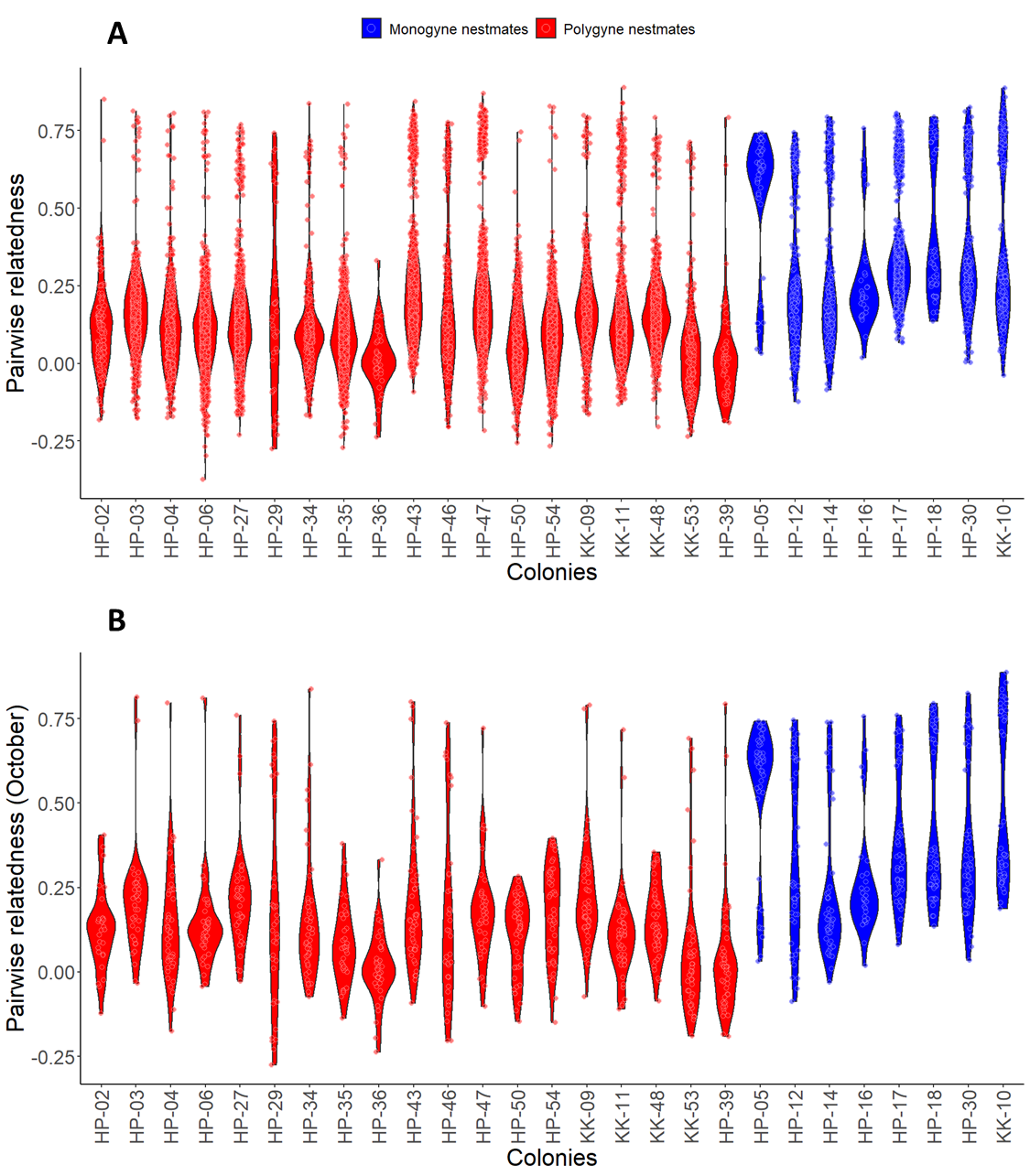


**Supplementary Figure 1.** Violin plots of within colony pairwise relatedness among workers collected along all the sampling periods (Oct-Jan) (A) and collected only in October (B). We observe a smaller number of siblings in polygyne colonies (in red) than in monogyne (in blue), although some full-siblings are detected.


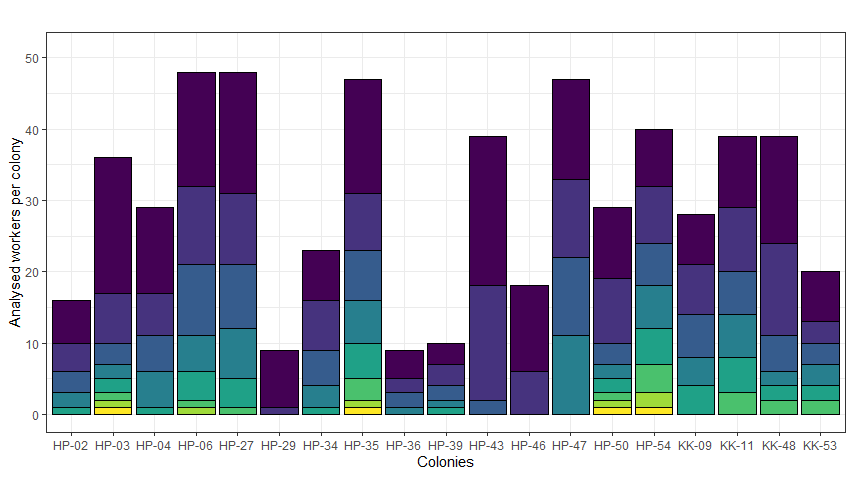


**Supplementary Figure 2.** Queens’ contribution to worker production in the 19 polygyne colonies based on COLONY results. Each different color within the colony corresponds to a different queen.  Queens varied substantially in their contribution to worker production in most colonies.


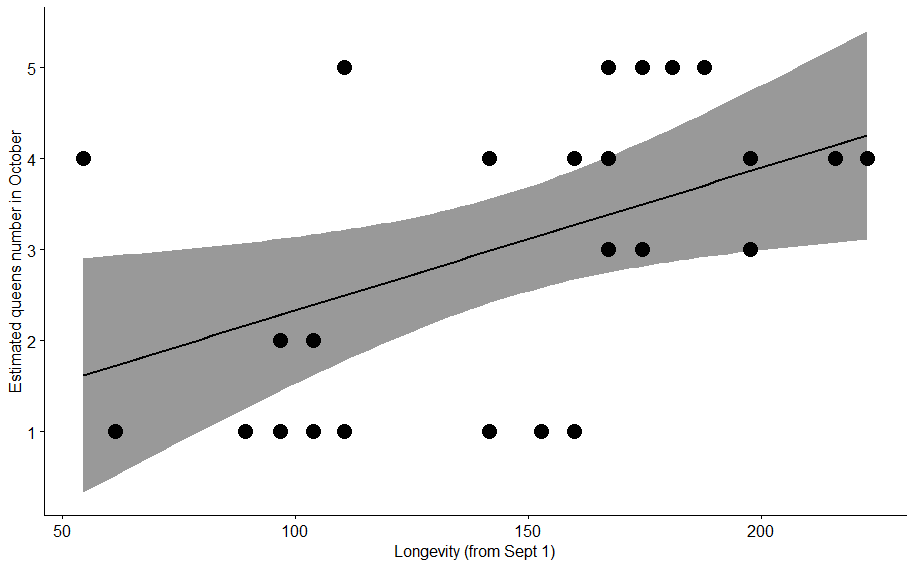


**Supplementary Figure 3.** Colonies that have greater numbers of queens already at the first sampling day appear to persist longer than colonies with one or two queens (Spearman correlation, R=0.49, p=0.01).


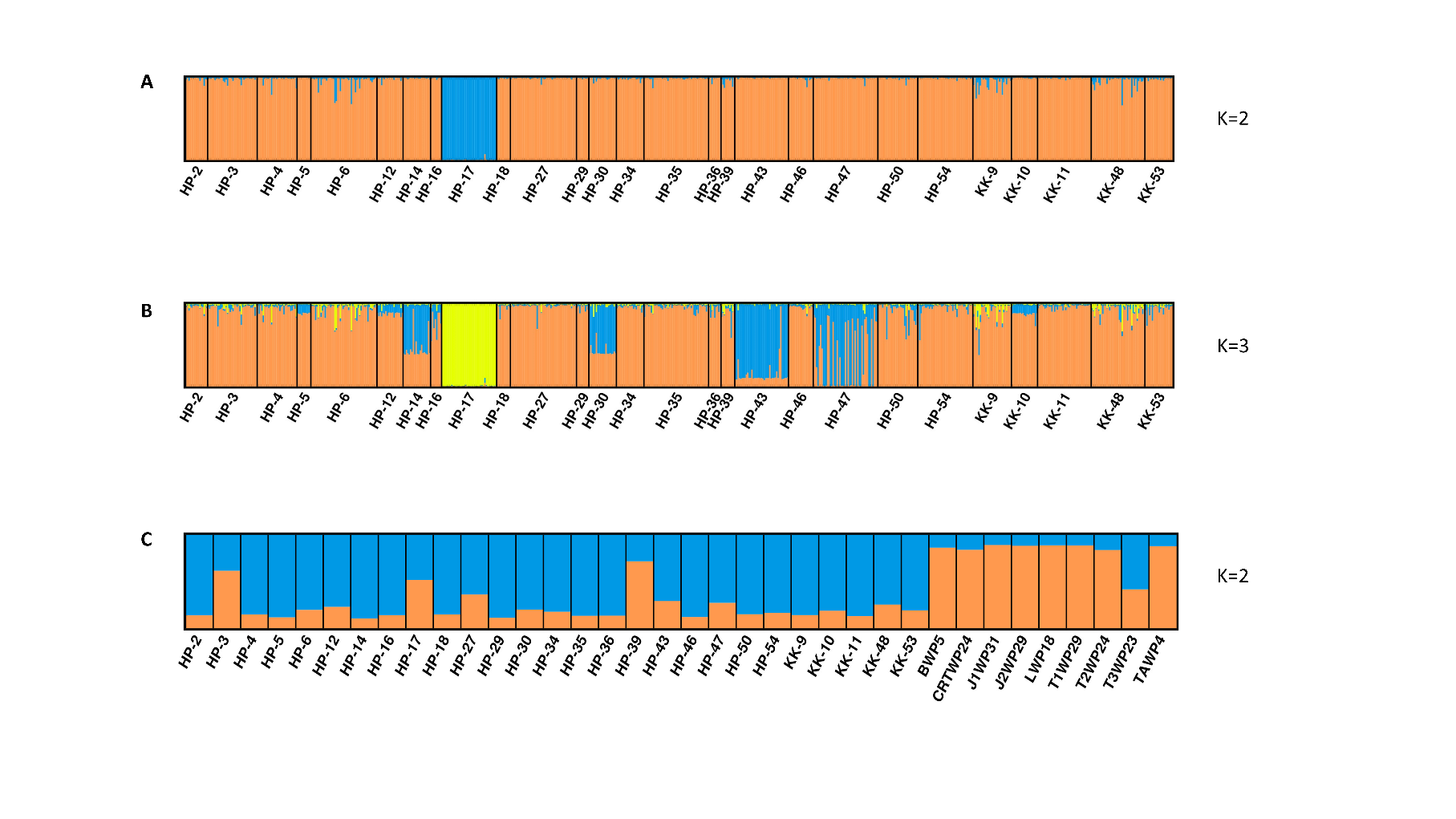


**Supplementary Figure 4.** We found no evidence of genetic structure among our 27 focal colonies within our study area based on a STRUCTURE analysis (K = 2) (A). Moreover, we found no difference in the population structure of monogyne and polygyne colonies, except for the colony HP-17 (monogyne) which appears differentiated from the others. The STRUCTURE result for K=3 is also shown (B). This pattern supports the assertion that queens (and possibly males) are not usually philopatric but instead may disperse over at least a kilometer scale. This distance is not surprising, given our observation that foragers will travel hundreds of meters multiple times per day to retrieve food. We observed more substantial genetic structure when we assessed our 27 focal colonies alongside nine colonies from more distant localities analyzed in Sankovitz et al (submitted) (C). This pattern suggests that the lack of population structure in our focal colonies does not solely reflect low genetic diversity in this invasive species. Instead, longer dispersal flights are probably relatively rare in the introduced *V. pensylvanica* population.
