# Supplementary material for: Early queen joining and long‐term queen associations in polygyne colonies of an invasive wasp revealed by longitudinal genetic analysis
Source: Evol Appl. 2021 Nov 30;14(12):2901–14. doi: 10.1111/eva.13324 (PMC8674895; doi:10.1111/eva.13324)
Supplement: Supplementary file 2 — Table S1 [file EVA-14-2901-s002.docx]

**Supplementary Table 1.** Overview of COLONY results across sampling dates per each of the 27 focal colonies. COLONY assigns a numerical name (e.g., #1, #2, #3) to each queen detected. The estimated number of queens and the names of the queens are shown.

|  |  |  | Oct 1 | | Oct 29 | | Nov 26 | | Dec 24 | | Jan 28 | |  |
| --- | --- | --- | --- | --- | --- | --- | --- | --- | --- | --- | --- | --- | --- |
| Latitude | Longitude | Colonies | Estimated queen # | Estimated queens | Estimated queen # | Estimated queens | Estimated queen # | Estimated queens | Estimated queen # | Estimated queens | Estimated queen # | Estimated queens | Total queens number |
| 19.345075 | -155.2666 | HP-02 | 4 | #1; #2; #3; #4 | 3 | #3; #4; #5 | - | - | - | - | - | - | 5 |
| 19.349331 | -155.2612 | HP-03 | 3 | #6; #7; #8 | 3 | #6; #12; #13 | - | - | 4 | #6; #7; #8; #9 | 4 | #6; #8; #10; #11 | 8 |
| 19.352104 | -155.2548 | HP-04 | 4 | #14; #15; #16; #17 | 4 | #14; #15; #16; #17 | - | - | 5 | #14; #15; #16; #17; #18 | - | - | 5 |
| 19.355798 | -155.2547 | HP-05 | 1 | #19 | - | - | - | - | - | - | - | - | 1 |
| 19.355148 | -155.2555 | HP-06 | 5 | #20; #21; #22; #23; #24 | 3 | #20; #22; #23 | 6 | #20; #21; #22; #23; #24; #25 | 6 | #20; #21; #22; #23; #24; #26 | 3 | #20; #22; #23 | 7 |
| 19.343925 | -155.2756 | HP-12 | 1 | #28 | 1 | #28 | - | - | - | - | - | - | 1 |
| 19.344344 | -155.2742 | HP-14 | 1 | #29 | 1 | #29 | - | - | - | - | - | - | 1 |
| 19.344674 | -155.2764 | HP-16 | 1 | #27 | - | - | - | - | - | - | - | - | 1 |
| 19.338373 | -155.2747 | HP-17 | 1 | #30 | 1 | #30 | 1 | #30 | 1 | #30 | - | - | 1 |
| 19.31888 | -155.2925 | HP-18 | - | - | 1 | #31 | - | - | - | - | - | - | 1 |
| 19.345123 | -155.2707 | HP-27 | 4 | #33; #34; #35; #37 | 2 | #32; #33 | 5 | #32; #33; #34; #35; #36 | 5 | #32; #33; #34; #35; #37 | 2 | #34; #37 | 6 |
| 19.353753 | -155.2528 | HP-29 | 2 | #38; #39 | - | - | - | - | - | - | - | - | 2 |
| 19.347641 | -155.2681 | HP-30 | 1 | #40 | 1 | #40 | - | - | - | - | - | - | 1 |
| 19.35051 | -155.2599 | HP-34 | 4 | #41; #42; #43; #44 | 5 | #41; #42; #44; #45; #46 | - | - | - | - | - | - | 6 |
| 19.363828 | -155.2488 | HP-35 | 4 | #47; #48; #49; #50 | 4 | #47; #48; #49; #53 | 5 | #47; #48; #49; #51; #52 | 3 | #49; #51; #53 | 6 | #48; #49; #51; #52; #53; #54 | 8 |
| 19.364252 | -155.249 | HP-36 | 4 | #55; #56; #57; #58 | - | - | - | - | - | - | - | - | 4 |
| 19.348985 | -155.2613 | HP-39 | - | - | 5 | #59; #60; #61; #62; #63 | - | - | - | - | - | - | 5 |
| 19.350912 | -155.2574 | HP-43 | 3 | #64; #65; #66 | 2 | #64; #65 | 2 | #64; #65 | 2 | #64; #65 | - | - | 3 |
| 19.335076 | -155.2784 | HP-46 | 2 | #67; #68 | 2 | #67; #68 | - | - |  | - | - |  | 2 |
| 19.335902 | -155.2775 | HP-47 | 4 | #69; #70; #71; #72 | 4 | #69; #70; #71; #72 | 4 | #69; #70; #71; #72 | 4 | #69; #70; #71; #72 | 3 | #69; #71; #72 | 4 |
| 19.3119 | -155.2977 | HP-50 | - | - | 3 | #77; #78; #79 | 4 | #77; #80; #81; #82 | 5 | #78; #80; #82; #83; #84 | - | - | 8 |
| 19.312391 | -155.2975 | HP-54 | - | - | 3 | #85; #86; #87 | 5 | #88; #89; #90; #91; #92 | 4 | #86; #90; #91; #92 | 4 | #88; #89; #90; #91 | 8 |
| 19.345068 | -155.2123 | KK-09 | 5 | #93; #94; #95; #96; #97 | 4 | #93; #95; #96; #97 | 5 | #93; #94; #95; #96; #97 | - | - | - | - | 5 |
| 19.344529 | -155.2118 | KK-10 | 1 | #98 | 1 | #98 | - | - | - | - | - | - | 1 |
| 19.34437 | -155.2139 | KK-11 | 5 | #99; #100; #101; #102; #103 | 4 | #99; #100; #102; #104 | 5 | #99; #100; #101; #103; #104 | 4 | #99; #100; #101; #104 | - | - | 6 |
| 19.341383 | -155.209 | KK-48 | 5 | #73; #74; #75; #76; #105 | 3 | #73; #74; #106 | 4 | #73; #74; #76; #105 | 4 | #73; #74; #75; #76 | - | - | 6 |
| 19.34753 | -155.2131 | KK-53 | 5 | #107; #108; #109; #110; #111 | 5 | #107; #109; #110; #111; #112 | - | - | - | - | - | - | 6 |
